# First purified recombinant CYP75B including transmembrane helix with unexpected high substrate specificity to (2*R*)-naringenin

**DOI:** 10.1038/s41598-022-11556-3

**Published:** 2022-05-20

**Authors:** Johanna Hausjell, Julia Weissensteiner, Christian Molitor, Karin Schlangen, Oliver Spadiut, Heidi Halbwirth

**Affiliations:** 1 Research Division Biochemical Engineering, Institute of Chemical, Environmental and Bioscience Engineering, Technische Universität Wien, Gumpendorfer Straße 1a, 1060 Vienna, Austria; 2 Research Division Bioresources and Plant Science, Institute of Chemical, Environmental and Bioscience Engineering, Technische Universität Wien, Getreidemarkt 9, 1060 Vienna, Austria

**Keywords:** Molecular biology, Biochemistry, Biocatalysis, Enzymes

## Abstract

Anthochlor pigments (chalcones and aurones) play an important role in yellow flower colourization, the formation of UV-honey guides and show numerous health benefits. The B-ring hydroxylation of chalcones is performed by membrane bound cytochrome P450 enzymes. It was assumed that usual flavonoid 3′-hydroxlases (F3′Hs) are responsible for the 3,4- dihydroxy pattern of chalcones, however, we previously showed that a specialized F3′H, namely chalcone 3-hydroxylase (CH3H), is necessary for the hydroxylation of chalcones. In this study, a sequence encoding membrane bound CH3H from *Dahlia variabilis* was recombinantly expressed in yeast and a purification procedure was developed. The optimized purification procedure led to an overall recovery of 30% recombinant *Dv*CH3H with a purity of more than 84%. The enzyme was biochemically characterized with regard to its kinetic parameters on various substrates, including racemic naringenin, as well as its enantiomers (2*S*)-, and (2*R*)-naringenin, apigenin and kaempferol. We report for the first time the characterization of a purified Cytochrome P450 enzyme from the flavonoid biosynthesis pathway, including the transmembrane helix. Further, we show for the first time that recombinant *Dv*CH3H displays a higher affinity for (2*R*)-naringenin than for (2*S*)-naringenin, although (2*R*)-flavanones are not naturally formed by chalcone isomerase.

## Introduction

**Figure 1:**
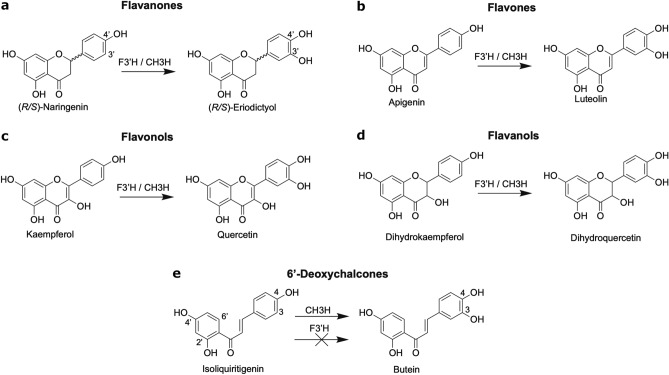
Reactions catalysed by chalcone 3-hydroxylase and flavonoid 3′-hydroxylase.

Dahlia (*Dahlia variabilis* hort.) is a popular ornamental plant exhibiting a broad spectrum of flower colour from all hues of red, magenta and orange, to yellow and white^1–3^. The great range of colours, as well as flower shapes, is presumably a result of the octoploidy of garden dahlia (2n = 8x = 64), allowing a multitude of gene combinations of the two tetraploid parent species^4^. In contrast to many other members of the Asteraceae species, in which carotenoids contribute significantly to yellow hues, Dahlia flower colour is exclusively based on the accumulation of flavonoids and biochemically related compounds^3^ and in the specific case of yellow flowered dahlia the glucosides of the 6′-deoxychalcones, isoliquiritigenin (2′,4′,4-trihydroxychalcone) and butein (2′,4′,3,4-tetrahydroxychalcone) are responsible^5,6^. All flavonoids have a C6–C3–C6 skeleton, which is synthesized from phenylpropanoid and polyketide precursors. Chalcone synthase (CHS) catalyses the committed step in flavonoid biosynthesis forming naringenin chalcone from (hydroxyl-)cinnamoyl-CoA and malonyl-CoA. In most cases Naringenin chalcone is then converted to (2*S*)-flavanones, catalysed by the chalcone isomerase (CHI). 6′-Deoxychalcones, in contrast, are synthesized by the co-action of the chalcone reductase (CHR; chalcone polyketide reductase, PKR) with CHS^7,8^. Flavonoid 3′-hydroxylation is performed by flavonoid 3′-hydroxlase (F3′H), which shows a broad substrate specificity and is able to hydroxylate flavanones (Fig. 1a), flavones (Fig. 1b), flavonols (Fig. 1c) and flavanols (Fig. 1d).

In contrast to the well understood pathway to red anthocyanin and ivory flavone pigments in dahlia, the biosynthesis of yellow anthochlors remains somewhat of an enigma with regard to the involved enzymes and genes^1,9,10^. For a long time, it was assumed that F3′H is responsible for the formation of the 3,4-dihydroxy pattern of chalcones, as it shows high similarity to the flavonoid 3′-hydroxylation reaction catalysed by F3′H. However, we have shown that chalcone 3-hydroxylation is not catalysed by F3′Hs^11^ (Fig. 1e). Specific structural prerequisites can increase the chalcone acceptance of single F3′Hs to some extent^12^, but a cDNA sequence encoding an enzyme with particularly high chalcone 3-hydroxylase (CH3H) activity was detected in yellow flower petals of *Cosmos sulphureus*^13^. It can be assumed that CH3H probably evolved in the Asteraceae family from F3′Hs to specifically attract insects that favour yellow and blue over bright red flower colour^14^. Studies with the CH3H enzyme of *Cosmos sulphureus* (*Cs*CH3H) showed that, besides chalcones, (2*S*)-naringenin, dihydrokaempferol, apigenin and kaempferol are also accepted as substrates^13^. Therefore, one can conclude that CH3H is a specialized F3′H as it exhibits an additional functionality compared to F3′H. Furthermore, it was shown that apple plants overexpressing *Cs*CH3H exhibited an in increased level of 3-hydroxyphloretin^15^, while the in vitro assays of apple F3′Hs did not show dihydrochalcone hydroxylation activity^16,17^.

CH3H and F3′H belong to the large superfamily of cytochrome P450 dependent monooxygenases (CYP). Cytochrome P450 genes are subdivided and classified on the basis of amino-acid identity, phylogenetic criteria and gene organization^18^. Plant CYPs are heme containing proteins and cluster in two clades, where one clade frequently has conserved metabolic housekeeping functions and is related to CYPs of other phyla, whereas the other one is plant specific and involved in secondary metabolism^19^. CH3H and F3′H cluster in the CYP75B subfamily. The CYP75 gene family is divided into two subfamilies and belongs to clan71^20^. It comprises the flavonoid biosynthetic enzymes, which are involved in the biosynthesis of the majority of plant secondary metabolites^21,22^. The subfamily CYP75A contains flavonoid 3′ 5′-hydroxylases (F3′5′Hs) and the subfamily CYP75B contains F3′H as well as CH3H enzymes^21,23^. Most F3′5′Hs belong to the CYP75A subfamily, with some exceptions that cluster into the CYP75B subfamily and have also gained 5′-hydroxylase acitivity^24–27^. Whereas prokaryotic CYPs are usually soluble proteins, plant CYPs are typically bound by their N-terminal single transmembrane domain^18^ either at the cytosolic face of the endoplasmic reticulum, or the inner mitochondrial membranes, or the plastids^19^. Further, both mammalian and plant CYPs need a redox partner, the NADPH dependent cytochrome P450 reductase (CPR). CPRs are FAD- and FMN-containing enzymes and are also membrane anchored at the cytosolic face of the endoplasmic reticulum^28^. In the flavonoid pathway, several steps are catalysed by CYP enzymes, with F3′H being the most prominent^29^. The association with the endoplasmic reticulum membrane makes these enzymes more difficult to handle in every aspect, from enzyme preparation and characterization, production and purification of recombinant enzymes, to structural studies^30^. In this study we report for the first time the isolation of a cDNA sequence encoding CH3H from dahlia flowers and the production, purification and characterization of the recombinant enzyme possessing its transmembrane helix. Further, we can report for the first time that the hydroxylation of (2*R*)-naringenin is catalysed by CH3H with a high substrate specificity, even though (2*R*)-naringenin is not naturally formed by CHI.

## Results and discussion

### Cloning and sequence analysis of the *Dv*CH3H

**Figure 2:**
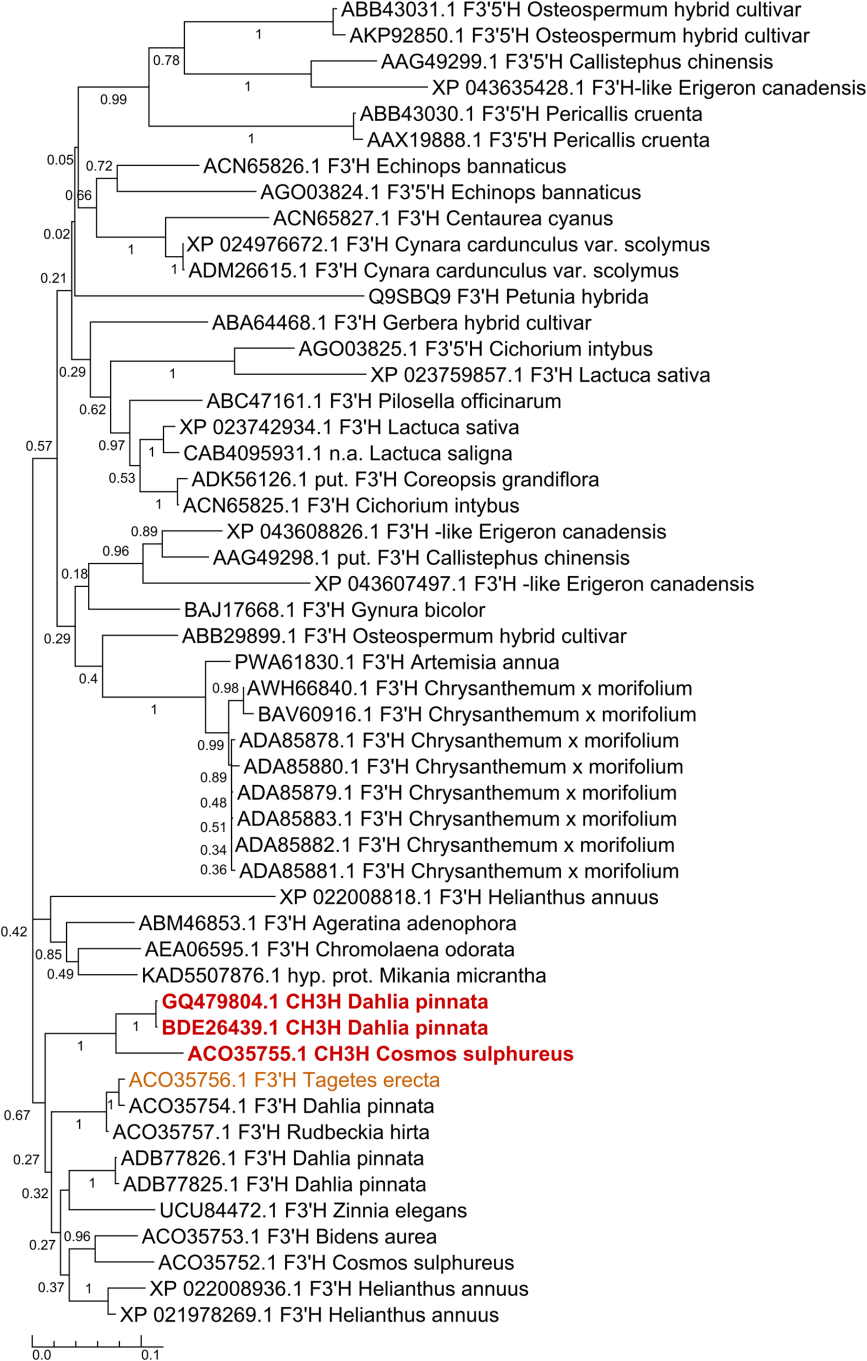
Phylogenetic tree of available Asteraceae CYP75B amino acid sequences. The enzymes exhibiting chalcone hydroxylase activity are coloured red and the F3′H from *Tagetes erecta* showing weak chalcone hydroxylase activity is coloured orange. Note: The sequence of F3′H from *Petunia hybrida* (Q9SBQ9) has been included for comparison with F3′H from non-Asteraceae species.

**Figure 3:**
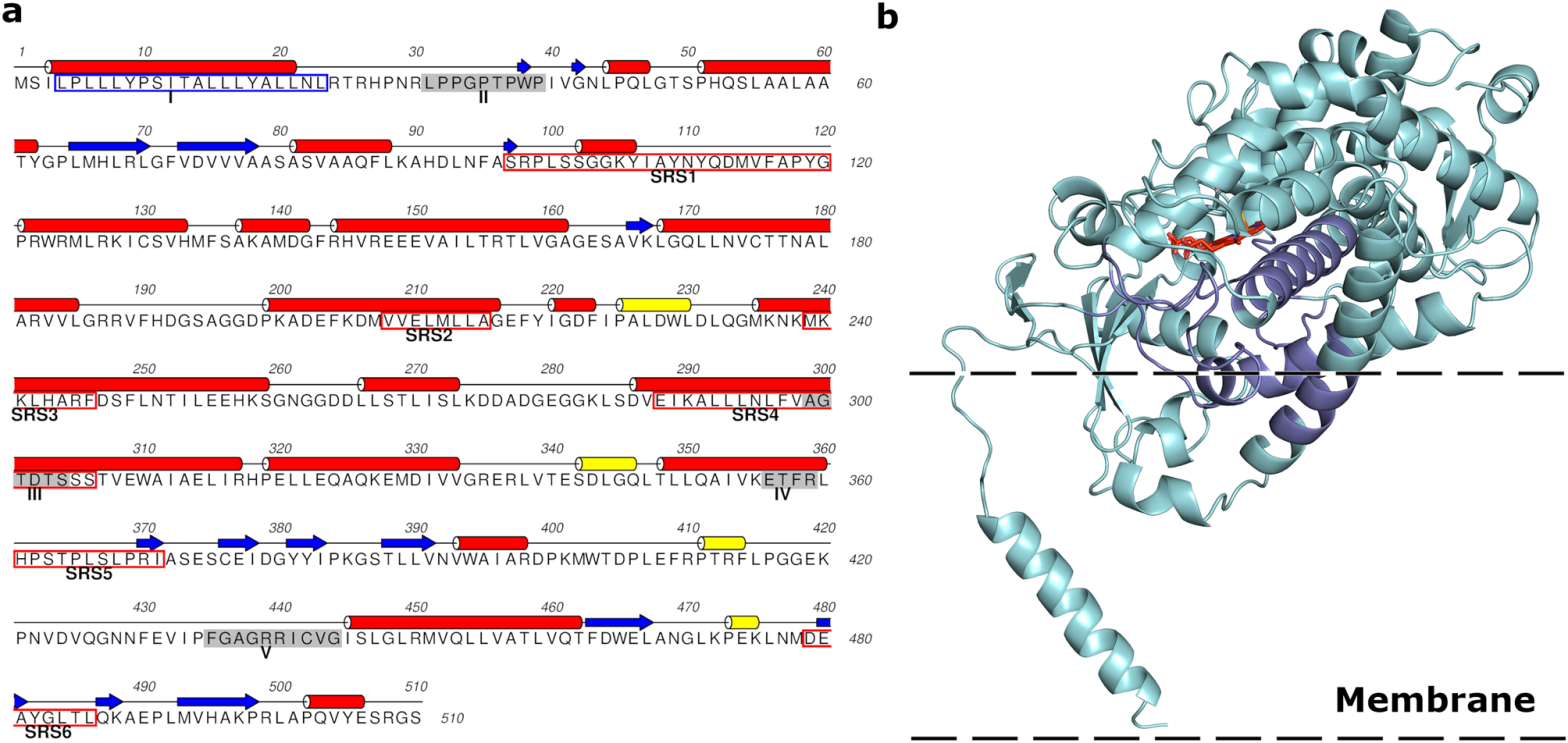
Structural features of *Dv*CH3H. (**a**) Sequence of *Dv*CH3H. Secondary structure features were assigned using DSSP (Dictionary of Secondary Structures of Proteins)^35^ using the homology model of *Dv*CH3H. Grey-shaded boxes highlight II, proline-rich hinge^13,36^; III, oxygen-binding motif; IV, EXXR triad^13,37^; V, the P450-pattern^13,38^. The blue framed box highlights the hydrophobic membrane anchor^13^ and the red framed boxes highlight the substrate recognition sites 1–6 (SRS1-6)^32–34^. **(b)** Homology model of *Dv*CH3H. The SRS regions is colored dark blue and the heme is coloured red. The putative orientation in and on the membrane of the endoplasmic reticulum has been roughly estimated according to the discussion in the literature^39^.

The cDNA from *D. variabilis (Dahlia pinnata)* encoding CH3H (deposited at the GenBank data library under accession number GQ479804) possesses an open reading frame of 1524 nucleotides, which was isolated with a degenerated primer designed in the 5′-noncoding region of the *Cosmos sulphureus CH3H*. The amino acids 4–23 of the deduced amino acid sequence are predicted by the program TMHMM^31^ to constitute the membrane anchor, which is attached to the membrane of the endoplasmic reticulum (Fig. 3). In order to obtain further information on the relationship of *Dv*CH3H to other CYP75B sequences within the Asteraceae family, a BLASTP search using the NCBI database was performed and 50 sequences were obtained after filtering. *Dv*CH3H shows a sequence identity of 70–84% to most of the obtained sequences (see Supplementary Fig. S1), while it shows 90% identity with *Cs*CH3H and 84–87% sequence identity with F3′Hs from *Tagetes, Dahlia, Rudbeckia, Zinnia, Bidens, Cosmos* and *Helianthus* (see Supplementary Table S1). This is also reflected by the corresponding phylogenetic tree (Fig. 2). The enzymes exhibiting chalcone hydroxylase activity, namely the two CH3Hs from *Dahlia pinnata* (GQ479804 and BDE26439) cluster together with *Cs*CH3H (ACO35755.1). The sequence BDE26439.1 has been released during the review process of this manuscript and is almost identical to GQ479804, with just two mutations within the membrane anchor and missing the last two amino acids of GQ479804. Notably, no other sequence clusters within this branch. However, it has been reported that F3′H from *Tagetes erecta* (ACO35756.1) showed weak chalcone hydroxylase activity in in vitro activity assays^11^, suggesting that the enzymes of this main branch might possess at least some of requirements necessary for chalcone hydroxylation. The substrate specificity of a CYP is determined by the amino acids within 6 substrate recognition sites (SRS)^32–34^ (Fig. 3) which are located in the proximity of the heme centre. In *Dv*CH3H the typical **LS**XX**G** pattern is present in SRS1, which is presumably required for the hydroxylation of chalcones, as well as the loop region found in *CsCH3H* (FJ216429)^13^.

### Purification

**Figure 4:**
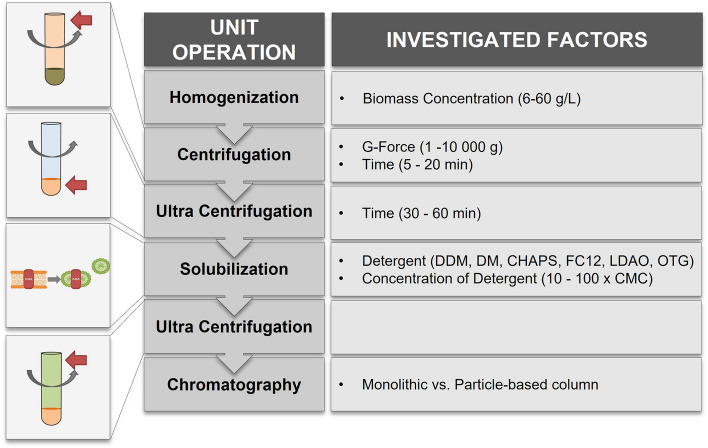
Unit operations necessary for the purification of recombinant *Dv*CH3H. Investigated factors and ranges thereof are shown in the right column.

A purification procedure for the membrane-bound *Dv*CH3H, recombinantly cultivated in controlled bioreactor runs in *P. pastoris* KM71H (for more details see^40^), was established by systematic investigation of each purification step. An overview is shown in Fig. 4.

### Homogenization and centrifugation

For yeast cell disruption, it has been reported that mechanical cell disruption techniques such as high pressure homogenization, bead milling or sonication lead to higher recovery yields, when it comes to breakage of the fungal cell wall, in comparison to electrical, enzymatic, physical or chemical disruption methods^41^. We chose high pressure homogenization, as the technique is efficient, scalable and results are reproducible. Previous studies on cell disruption of yeast by high pressure homogenization showed that the number of passages and the pressure had the most significant impact^42^. Therefore, we kept those parameters high (1800 bar and 10 passages) and investigated the impact of the dry cell weight concentration on the homogenization process. The dry cell weight is taken for the calculation of the used biomass as it is much more accurate than the wet cell weight and necessary for the optimization of the homogenization process. For the wet cell weight, the remaining water in the cell pellet depends a lot on the centrifugation process and strongly biased the calculation of the used cell mass. Samples were homogenized at dry cell weight concentrations between 6 and 60 g/L and centrifuged at 20,000× g for 30 min afterwards. As shown in Table 1, there is a slight trend towards less product in the supernatant at higher biomass concentrations. However, as the recovery of target protein at 60 g DCW/L was only 6% lower than at 6 g DCW/L, we decided to persist with the high biomass concentration, as this allowed working with a more concentrated protein solution, reducing the volumes during subsequent purification steps. Nevertheless, in general, around 60% of the target protein was found in the cell pellet, which is discarded after the disruption procedure. We hypothesized that this could result from (I) poorly disrupted cells or (II) a too harsh centrifugation afterwards, which caused partial sedimentation of the target protein. To invesigate the disruption efficiency, colony forming units (CFUs) of frozen and thawed cells were compared to frozen, thawed as well as homogenized cells and we found that the homogenization process only led to a reduction of 55% of CFUs. However, due to cooling limitations, neither the pressure nor the number of passages could be further increased.

**Table 1:** Results of western blots with percentages of recombinant *Dv*CH3H in supernatants (S) and pellets (P).

**Homogenization**												
DCW (g/L)	6		19.5		33		46.5		60			
Fraction	P	**S**	P	**S**	P	**S**	P	**S**	P	**S**		
% of *Dv*CH3H	55	**45**	56	**44**	63	**37**	66	**34**	60	**40**		
**Centrifugation**												
Force (g)	1,000		1,000		10,000		10,000		5,500			
Time (min)	5		20		5		20		12.5			
Fraction	**S**	P	**S**	P	**S**	P	**S**	P	**S**	P		
% of *Dv*CH3H	**65**	35	**73**	27	**51**	49	**56**	44	**53**	47		
**Ultra-Centrifugation**												
Time (min)	30		60									
Fraction	**P**	S	**P**	S								
% of *Dv*CH3H	** > 99**	< 1	** > 99**	< 1								
**Solubilization—detergent type**												
Detergent	DDM		DM		CHAPS		FC-12		LDAO		OTG	
Fraction	**S**	P	**S**	P	**S**	P	**S**	P	**S**	P	**S**	P
% of *Dv*CH3H	**27**	73	**18**	82	** < 1**	> 99	** < 1**	> 99	**36**	64	**12 + 65**^a^	23
**Solubilization – detergent concentration**												
DDM	20 mM											
Fraction	**S**	P										
% of *Dv*CH3H	**61**	39										

The fractions that were further processed are marked in bold.

DDM: n-Dodecyl β-D-maltoside; DM: n-Decyl-β-D-maltoside; CHAPS: 3-((3-Cholamidopropyl) dimethylammonio)-1-propanesulfonate; FC-12: Fos-choline-12; LDAO: Dodecyldimethylaminoxid; OTG: Octylthioglucoside.

^a^OTG fractionated the protein during SDS-PAGE, therefore two different bands were detected on the Western blot. The 12% band was at the correct size whereas the 65% band was much smaller. Average standard deviation of the results is 6.4%. Western blots for the homogenisation and the detergent type are provided in Supplementary Fig. 2.

Next, it was investigated whether a less harsh centrifugation procedure could lead to higher amounts of target protein in the supernatant. In literature, the seperation of cell debris is usually carried out by centrifugation between 1000 and 15,000× g for 15 min up to 30 min, which is a rather broad range^43–47^. To shed more light on the optimal conditions, g-forces between 1000 and 10,000, and centrifugation times between 5 and 20 min were investigated. The results in Table 1 show that a reduction in the g-force clearly leads to more protein in the supernatant with a recovery of up to 73%. At the same time, it was beneficial to keep the centrifugation time rather high. This can be explained as with shorter centrifugation times, the pellet is more voluminous, causing a lower recovery of target protein in the supernatant. Further reduction of the centrifugation force led to an incomplete separation of unbroken cells.

### Ultracentrifugation

After cell disruption, the membrane protein fraction was collected by ultracentrifugation. It is generally recommended to pellet membrane proteins at 100,000–200,000× g for 1- 2 h^43–45,48^. We investigated the time needed to pellet recombinant *Dv*CH3H at 200,000× g by analysing samples after 30 min and 60 min. As shown in Table 1, already after 30 min, no target protein was detectable in the supernatant anymore. Longer centrifugation times produced a more compact pellet which was more difficult to resuspend and solubilize in the subsequent step. Therefore, 30 min at 200,000× g was chosen for subsequent experiments.

### Solubilization and ultracentrifugation

After pelleting the membrane protein fraction, the target protein needs to be solubilized out of the membrane by detergents. Therefore, we tested solubilisation in six different detergents at 4 °C over night^45^, always at 10 times their critical micellar concentration. The obtained results are shown in Table 1. LDAO solubilized the highest amount of target protein (36%), however, initial purification results showed that the protein was very unstable in this detergent. LDAO is considered a rather harsh detergent due to its zwitterionic nature as its charges can interact with non-hydrophobic parts of the protein potentially causing instability issues. OTG seemed to have a negative influence on protein stability, as a band of very low molecular weight was visible on the blot, which might originate from *Dv*CH3H (see Supplementary Fig. S2b). Possible causes of this potential cleavage would have to be investigated in detail, however, exceed the scope of the manuscript. The detergents CHAPS and FC-12 did not solubilize a detectable amount of protein. DDM solubilized the second highest amount of target protein (27%) which is why this detergent was chosen for further experiments. Adjustment of the solubilisation time to 4 h, the detergent concentration to 20 mM and the protein concentration to 2 mg/mL allowed the extraction of 61% of the target protein (Table 1).

### Chromatography

To further purify the recombinant transmembrane possessing his6-tagged *Dv*CH3H, the solubilized membrane protein solution was loaded on immobilized metal ion affinity chromatography (IMAC) columns. Affinity chromatography can be quite challenging for tagged membrane proteins, as they sometimes bind very poorly to the resin due to the huge detergent micelles, which impair accessibility of the tag^45^. In order to reduce the detergent concentration in the load, the solubilized protein was diluted to a final DDM concentration to 2 mM with detergent free buffer. We were interested in whether a classic particle-based column or a monolithic column was superior for the purification. The results of two purification runs, one on a particle-based column and one on a monolithic column, with identical column volumes and similar metal ion capacities (15 µmol Ni^2+^/mL resin (particle based) and 23 ± 10 µmol Cu^2+^/mL resin (monolithic)) are compared in Table 2.

**Table 2:** Comparison of the IMAC purification on a monolithic and particle-based column.

Column	Purity (%)	Purification factor (–)	Recovery of *Dv*CH3H (%)
Particle based	56 ± 0.6	43 ± 26	93 ± 28
Monolithic	32 ± 0.3	25 ± 15	71 ± 23

**Figure 5:**
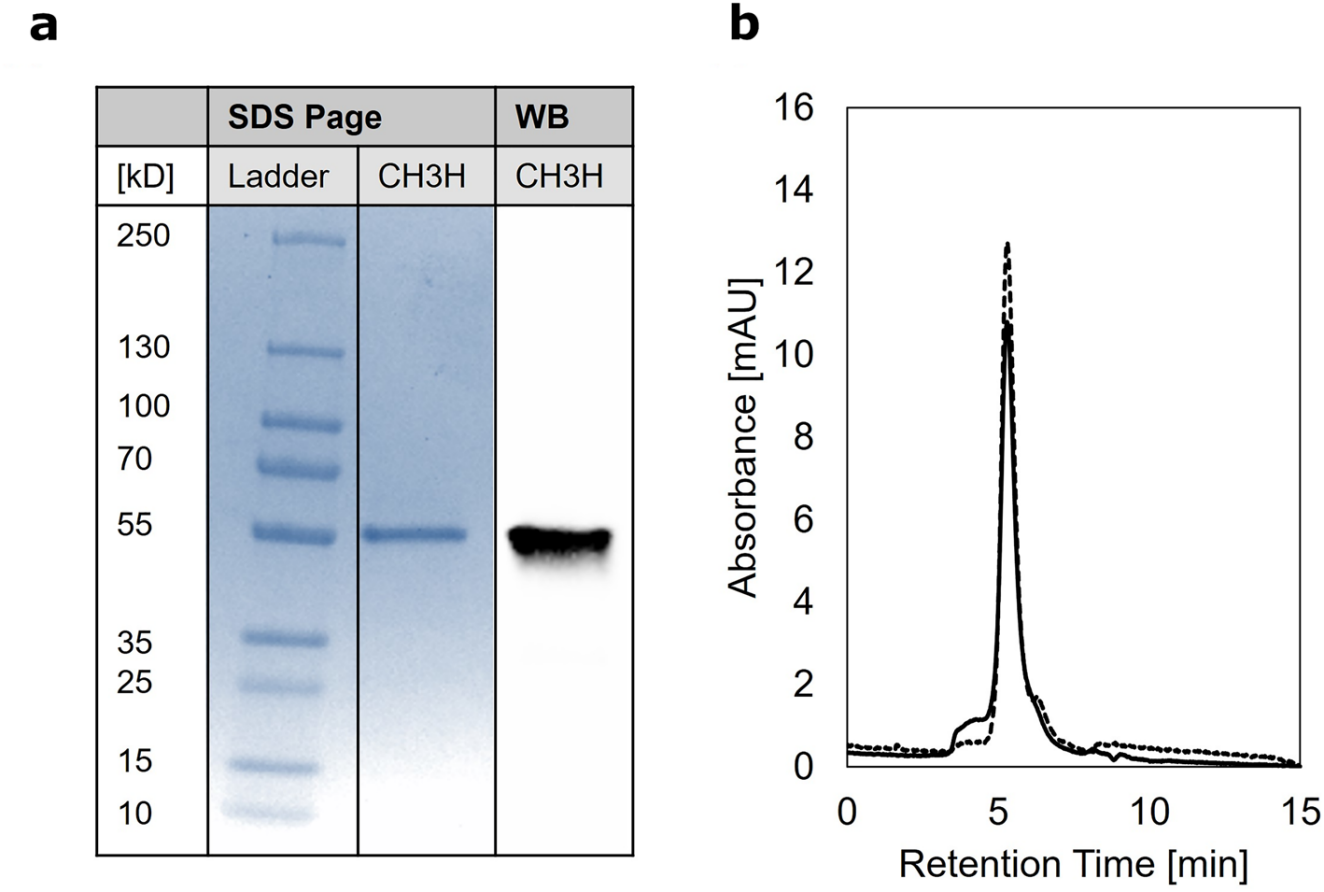
(**a**) SDS-PAGE and western blot and (**b**) SEC-chromatogram of the purified recombinant *Dv*CH3H. Solid line indicates absorbance at 280 nm and the dashed line the absorbance at 425 nm, where the heme of the Tris-(2-carboxyethyl)-phosphin reduced recombinant *Dv*CH3H has an absorbance maximum. The original SDS-PAGE and western blot are shown in Supplementary Fig. S3.

After 1:10 dilution of the load, rather high recoveries of *Dv*CH3H were found, especially on the particle-based column, where 93% could be captured and eluted. On the monolithic column in contrast only 71% of *Dv*CH3H were recovered in the eluate and binding of proteins in general seemed less specific as the purity of the eluate was only 32%. However, the eluate of the particle-based column was also only 56% pure, which is why an additional size exclusion chromatography was carried out. This allowed purification to more than 84%, as determined by analytical size exclusion chromatography (Fig. 5b), SDS-PAGE and Western blot (Fig. 5a).

### Overall purification process

The entire purification process is summarized in Table 3. Values marked with an asterisk were theoretically calculated based on indirect measurements, when direct determination was not possible (for details see SI).

**Table 3:** Overview of the purification process.

	Protein Concentration (mg/ml)	Relative Specific Activity (% of purified *Dv*CH3H)	Recovery (%)	Purification factor (–)
Homogenization and Centrifugation	13.6 ± 0.30	0.59 ± 0.08	73 ± 4	–
Ultra Centrifugation	5.7 ± 0.30	1.38 ± 0.23	99 ± 5	2.3^a^ ± 0.3
Solubilization and Ultra Centrifugation	2.1 ± 0.10	1.54^a^ ± 0.75	61 ± 3	1.1^a^ ± 0.6
IMAC Particle-based Column	1.35 ± 0.02	66.7^a^ ± 18	93^a^ ± 28	43^a^ ± 26
SEC	0.22 ± 0.01	100	73^a^ ± 10	1.5^a^ ± 0.4
Overall			30^a^ ± 18	170^a^ ± 23

^a^Theoretical values were calculated from indirect measurements, as indicated in the text, because direct measurements were not possible.

The overall recombinant *Dv*CH3H recovery was 30%, with over 60% in each of the five purification steps. The most critical step was the solubilisation of the membrane protein. Advanced optimization with respect to the ionic strength or the pH of the solubilisation buffer might result in higher yields^45^.

The overall purification factor was 170, leading to at least 84% pure recombinant *Dv*CH3H, as judged by analytical HPLC-SEC. This protein fraction was used for biochemical characterization of *Dv*CH3H.

### Biochemical characterization

#### Kinetic properties of recombinant *Dv*CH3H

*Dv*CH3H needs a cytochrome P450 reductase (CPR) as redox partner in order to be catalytically active. Therefore, microsomal preparations of yeast cells expressing *Catharanthus roseus* NADPH-cytochrome P450 reductase (*Cr*CPR) were included in all activity assays. In the presence of microsomal preparations of recombinantly expressed *Cr*CPR and NADPH as the electron donor, recombinant *Dv*CH3H converted isoliquiritigenin to butein, naringenin to eriodictyol, apigenin to luteolin, kaempferol to quercetin, and dihydrokaempferol to dihydroquercetin. However, isoliquiritigenin and dihydrokaempferol were not only metabolised by *Dv*CH3H to butein and dihydroquercetin, but also by yeast originating enzymes present in the microsome preparation of recombinant *Cr*CPR to unidentified reaction products. Due to the high extent of these unknown products, the kinetic parameters of these substrates would be strongly biased and, therefore, could not be included in the kinetic studies.

For the other substrates, the pH optimum of the recombinant *Dv*CH3H was investigated from pH 5.5 to pH 8.5 (see Supplementary Fig. S4). The pH optimum of *Dv*CH3H for all tested substrates is between 7.0 and 8.0. *Dv*CH3H shows the highest activity for naringenin between pH 7.0 and pH 7.5, for kaempferol between pH 7.5 and pH 8.0 and for apigenin at pH 7.5. For better comparability, the kinetic measurements were performed at pH 7.5. A NADPH concentration of 1.55 mM showed a sufficient excess, without limiting the activity of *Dv*CH3H.

**Figure 6:**
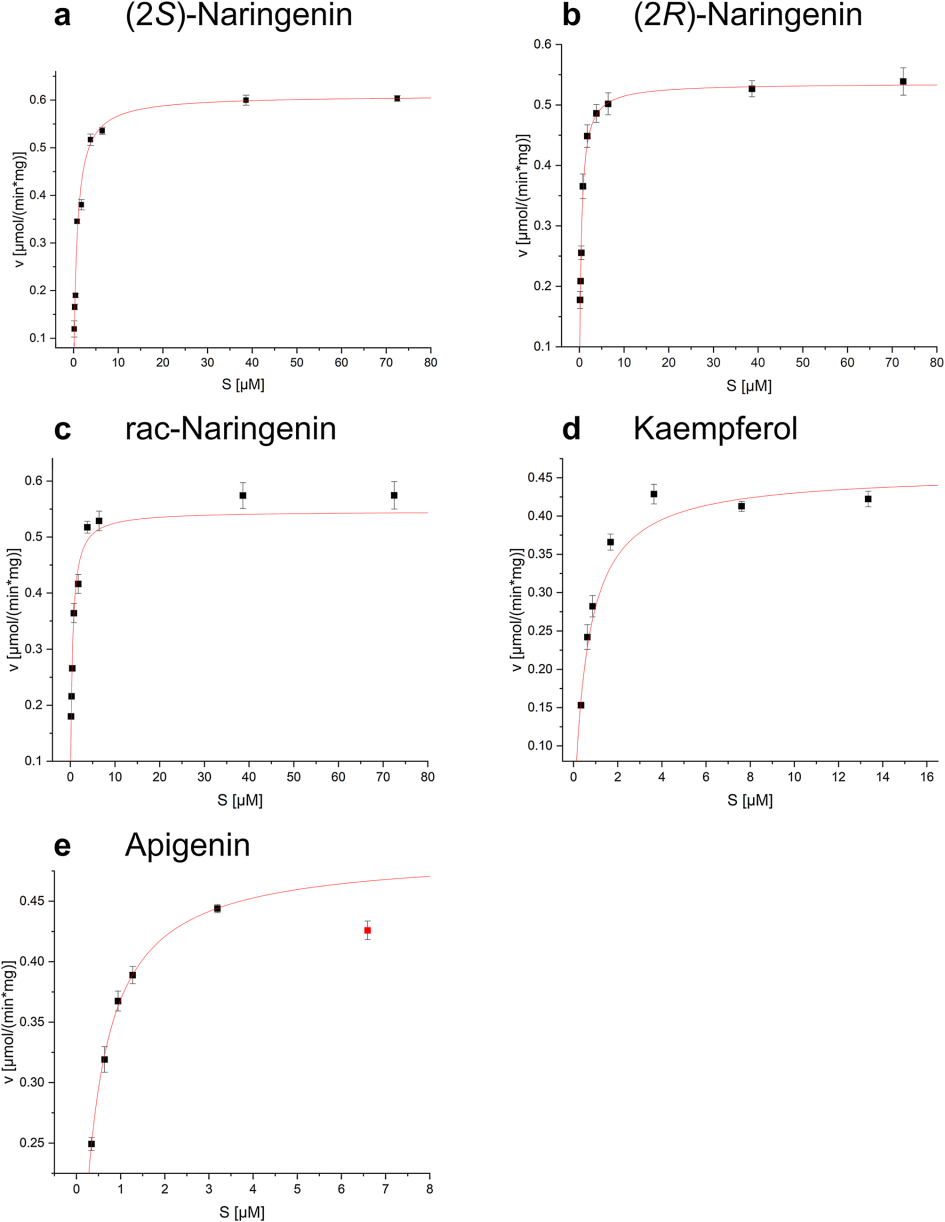
Specific activity (v) of the recombinant membrane bound *Dv*CH3H in dependence of the substrate concentration (S). The graphs show steady state kinetic measurements of *Dv*CH3H using (**a**) (2*S*)-naringenin, (**b**) (2*R*)-naringenin, (**c**) racemic naringenin, (**d**) kaempferol and (**e**) apigenin as substrates.

Kinetic analysis of recombinant *Dv*CH3H was performed with the flavonol kaempferol, the flavone apigenin, and the flavanone naringenin. Usually, just a racemic mixture of naringenin is used as a substrate for investigations of enzymes involved in the flavonoid pathway. In the flavonoid pathway, however, only the (2*S*)-enantiomer is formed by CHI. A racemic mixture can be formed in planta, if the chalcones are isomerized chemically and in the absence of CHI. In this study, we therefore tested the racemic mixture and the pure enantiomers separately. The substrate concentration was varied in a range of 0.25–100 µM (Fig. 6) at a fixed concentration of 1.55 mM of NADPH, and the product formation was quantified by HPLC. The respective values for *K*_M_, v_max_ and k_cat_ were calculated from the curves in Fig. 6 and are summarized in Table 4.

**Table 4:** Kinetic parameters of purified recombinant membrane bound *Dv*CH3H.

Substrates	λ (nm)	*K*_M_ (µM)	v_max_ (µmol min^−1^ mg^−1^)	k_cat_ (s^−1^)	k_cat_/ *K*_M_ (µM^−1^ s^−1^)
(2*R*)-Naringenin	290	0.408 ± 0.015	0.536 ± 0.010	0.501 ± 0.01	1.228 ± 0.069
(2*S*)-Naringenin	290	0.76 ± 0.094	0.610 ± 0.025	0.571 ± 0.023	0.751 ± 0.124
Racemic Naringenin	290	0.402 ± 0.046	0.546 ± 0.031	0.511 ± 0.029	1.271 ± 0.219
Apigenin	346	0.329 ± 0.007	0.49 ± 0.002	0.458 ± 0.002	1.395 ± 0.033
Kaempferol	370	0.622 ± 0.069	0.457 ± 0.015	0.427 ± 0.014	0.688 ± 0.098

λ indicates the wavelength used for quantification of the reaction product.

The kinetic data for apigenin, kaempferol, (2*S*)-naringenin, (2*R*)-naringenin and the racemic naringenin show *K*_M_ values in a range below 1 µM, reflecting a high affinity of recombinant *Dv*CH3H to all substrates (Table 4). The lowest *K*_M_ value and the highest catalytic efficiency was observed with apigenin as substrate. In contrast, the highest *K*_M_ value was obtained with (2*S*)-naringenin as substrate, however, the catalytic efficiency was slightly higher than the catalytic efficiency for apigenin. Unexpectedly, recombinant *Dv*CH3H displayed a catalytic efficiency for (2*R*)-naringenin that is comparable to that of kaempferol. Furthermore, (2*R*)-naringenin showed a near two-fold higher affinity and a slightly higher turnover number than (2*S*)-naringenin, although it is not the naturally formed enantiomer in the flavonoid pathway.

The *K*_M_- and v_max_ value of the racemic naringenin basically reflects the values obtained with (2*R*)-naringenin, due to the significantly higher affinity and turnover rates of (2*R*)-naringenin in comparison to (2*S*)-naringenin. This indicates that recombinant *Dv*CH3H would preferentially transform (2*R*)-naringenin to (2*R*)-eriodictyol, if racemic mixtures would be naturally available as substrate. A stereospecific interaction of naringenin enantiomers with various CYPs has been previously reported and is of pharmacological interest as naringenin glycosides are abundantly present in grapefruit juice, which is known to inhibit metabolization of drugs^49^. Interestingly, (2*R*)-naringenin was previously shown to be converted by the recombinant 2-oxoglutarate dependent flavonol synthase from *Citrus unshiu* to (−)-*trans*-dihydrokaempferol, but the subsequent conversion into the corresponding flavonol was not possible^50^. As other enzymes, such as DFR and flavone synthase I, are also stereospecific^51^, (2*R*)-naringenin conversion does not seem to be of physiological relevance in the latter steps of the flavonoid pathway.

From the natural substrates, the flavone apigenin seems to be the preferred substrate. Compared with the kinetic data reported for CH3H of *Cosmos sulphureus*^12^, recombinant *Dv*CH3H shows a distinct difference in the flavonoid preferences. Recombinant *Dv*CH3H has the highest catalytic efficiency for apigenin, whereas recombinant *Cs*CH3H has the highest hydroxylation efficiency with kaempferol, apart from the preferred isoliquiritigenin. Unfortunately, chalcones could not be tested with the current test system. However, from the three flavonoid substrates tested, isoliquiritigenin, shows the closest structural similarity to apigenin due to the double bond in the C3-bond connecting rings A and B, and in contrast to the flavonol kaempferol, no hydroxyl group is present at the double bond. Based on these similarities, a high affinity for isoliquiritigenin and a high efficiency in conversion of this substrate can be hypothesized. Activity assays with purified CPR, as well as crystal structure analysis of substrate-enzyme complexes, will provide further insights into the differing binding modes of the different substrates.

## Conclusion

This is the first report of the isolation of a *CH3H* cDNA sequence of dahlia flowers, as well as the recombinant production, purification and biochemical characterization of the enzyme. We developed a purification procedure for the transmembrane helix possessing *Dv*CH3H by step-wise optimization of each downstream processing step. The final procedure allowed an overall recovery of 30%, with more than 60% in each purification step. The obtained *Dv*CH3H was 84% pure and biochemically characterized regarding its pH optimum, as well as kinetic parameters on various substrates, including apigenin, kaempferol and the enantiomers of naringenin. In particular, we were able to detect the hydroxylation of (2*R*)-naringenin, which is not the enantiomer that is naturally formed by CHI. The results potentially lay the groundwork for future crystallization of *Dv*CH3H, and thus understanding its ability to hydroxylate chalcones in position 3, a feature that is not inherent to the majority of F3′Hs.

## Materials and methods

### Plant material

The investigations were performed with petals of *D. variabilis* cv. Aurora’s kiss (Plant nursery Wirth, Vienna, Austria). The plant material was collected during the summers of 2007 and 2008, frozen in liquid nitrogen and stored at − 80 °C.

### Cloning and recombinant expression of *Dv*CH3H in *Pichia pastoris*

Extraction of mRNA and cDNA synthesis were performed with the µMACS mRNA isolation kit (Miltenyi Biotec, Bergisch Gladbach, Germany) and are described in detail in the SI and Supplementary Table S2. The cloning into the appropriate vectors, the transformation into *Pichia pastoris* as well as the recombinant expression were all performed as described previously^40^. The cultivated cells were harvested by centrifugation at 7000× g for 20 min at 4 °C, the supernatant was discarded and the cell biomass was frozen at − 20 °C until further use.

### Sequence and phylogenetic analysis

The amino acid sequence of *Dv*CH3H was used as a query for a BLASTP search in the NCBI database. The search was limited to Asteraceae species, the expect value was set to 1e-190 and the maximal target sequences was set to 250 sequences. The obtained sequences were aligned and partial sequences were omitted before the remaining 51 sequences were subjected to phylogenetic analysis by MEGA11 software^52^. The amino acid sequences were aligned using the muscle algorithm with default parameters followed by construction of a phylogenetic tree based on the maximum likelihood (Jones-Taylor-Thornton (JTT) model) with default parameters and 1000 bootstrap replicates.

#### Amino acid sequence diagram with secondary structure features

A homology model of *Dv*CH3H was prepared with MODELLER v10.2^53^ using the structure of CYP76AH1 (pdb: 5YM3) as well as the homology model of F3′H from *Petunia hybrida* (Q9SBQ9) obtained from the structure prediction website alphafold.ebi.ac.uk^54^. Molecular graphic images were generated with PyMOL (www.pymol.org) and secondary structure features were assigned using DSSP (Dictionary of Secondary Structures of Proteins)^35^.

### Cloning and heterologous expression of *Cr*CPR

The gene encoding a NADPH-dependent cytochrome P450 reductase *Cr*CPR (GenBank: X69791.1) was codon-optimized for expression by a commercial supplier (Genscript, Piscataway, NJ, USA). For details of cloning and heterologous expression in *Saccharomyces cerevisiae* see SI.

### Purification of transmembrane helix possessing *Dv*CH3H

#### Homogenization and microsomal preparation

Unless stated otherwise, homogenization was carried out as follows: 60 g dry cell mass were dissolved per 1 L buffer A (50 mM Tris, pH 8, 100 mM NaCl, 10% Glycerol, 0.5 mM TCEP) by mixing with an immersion blender type 4179 (Braun, Neu Isenburg, Germany). For the calculation of the dry cell mass an aliquot of the cell suspension was dried at 105 °C. The resuspended cells were then homogenized at 1800 bar for 10 passages with a PandaPLUS2000 homogenizer (GEA, Düsseldorf, Germany). The homogenized biomass was centrifuged at 1000 × g for 20 min at 4 °C in a type Z 36 HK centrifuge (Hermle, Wehningen, Germany) unless stated otherwise.

Ultracentrifugation was carried out with a Sorvall WX Ultra Series WX 80 centrifuge (Thermo Fisher Scientific, Waltham, MA, USA) using a fixed angle Type 50.2 Ti Rotor (Beckman Coulter, Brea, CA, USA). The supernatant of the homogenized and centrifuged sample was filled into Optiseal Polyallomer Tubes (Beckman Coulter, Brea, CA, USA) and ultracentrifuged at 4 °C for 30 min at 200,000 × g unless stated otherwise.

The analyses of the different steps were carried out as follows: For the homogenization process, frozen and thawed cells and frozen, thawed and homogenized cells (at the same dry cell weight concentration) were diluted 1:2 to 1: 1,000,000 with sterile homogenization buffer and then streaked on YPD-zeocin plates. The plates were incubated for 3 days at 30 °C before counting the CFUs. For the homogenization and centrifugation efficiency, the cell pellet and supernatant were diluted to the original volume with buffer A and then analysed by use of western blots.

#### Optimization of the membrane solubilisation

The list of the tested detergents is shown in Table 5. For membrane solubilisation the pellets were redissolved in 1.5 times the original volume with buffer A by mixing with an Ultra-Turrax IKA T10 basic Instrument (IKA, Staufen, Germany). Concentrated detergent stock solution was added to a final concentration of 10 × critical micelle concentration (CMC) for each of the detergents and the solution was swayed on a PMR-30 Compact Fixed-Angle Platform Rocker (Grant Instruments, Royston, UK) at 4 °C for 4 h unless stated otherwise. Then, the mixtures were subjected to ultracentrifugation at 200,000 × g for 30 min. The solubilization efficiency was determined by diluting pellet and supernatant to the original volume with buffer A and subjecting the samples to western blots.

**Table 5:** Detergents used for membrane solubilisation, including their abbreviations as used in the following, as well as suppliers.

Detergent	Critical micelle concentration in water (CMC) (mM)	Micelle MW (kDa)
n-Dodecyl β-D-maltoside (DDM, Thermo Fisher Scientific)	0.17; 0.12 (with 0.2 M NaCl)^a,^^b^	~ 72^b^
n-Decyl-β-D-maltoside (DM, Cube Biotech)	1.66^a^	~ 33 (69 molecules)^b^
3-((3-Cholamidopropyl) dimethylammonio)-1-propanesulfonate (CHAPS, Sigma-Aldrich)	4.2–6.3^a^	~ 6.2^c^
Fos-choline-12 (FC-12, Cube Biotech)	1.5^a^	~ 19 (54 molecules)^b^
Dodecyldimethylaminoxid (LDAO, Sigma Aldrich)	1–2^b^	~ 17–22^b^
Octylthioglucoside (OTG, Cube Biotech)	9 mM^a^	Unknown

References of the CMC-values and the micelle MW.

^a^cube-biotech.com.

^b^https://www.anatrace.com/.

^c^https://www.sigmaaldrich.com/.

#### Immobilized metal ion affinity chromatography

For preparative immobilized metal ion affinity chromatography (IMAC) an ÄKTA pure system (GE, Boston, MA, USA) was used. The supernatant of the detergent treated microsomes was diluted 1:10 with buffer A to reduce the detergent concentration. Additionally, a 500 mM imidazole solution in buffer A was added to reach a final concentration of 20 mM imidazole in the sample. The solution was loaded onto a pre-equilibrated column (either HisTrap FF column (GE, Boston, MA, USA) or CIM IDA-1 (BIA Separations, Ljubljana, Slovenia), both loaded with nickel ions) with a flow-rate of 127.5 cm/h. Elution was performed in a step gradient to 100% buffer B (50 mM Tris, pH 8, 100 mM NaCl, 10% glycerol, 0.5 mM TCEP, 500 mM imidazole, 2 mM DDM).

#### Preparative and analytical size exclusion chromatography

Preparative Size Exclusion Chromatography was carried out with a pre-equilibrated HiLoad 16/600 Superdex 200 pg (GE, Boston, MA, USA) for further purification. Analytical size exclusion chromatography was realized on an Ultimate HPLC 5000 (Thermo Scientific, Waltham, MA, USA) with a Superdex 200 Increase 5/150 GL (GE, Boston, MA, USA) (for details see SI).

#### Protein concentration determination SDS-PAGE and western blot

The protein concentration was measured by Bradford assays in crude samples (for details see SI). SDS-PAGE and western blot analysis are described in detail in the SI.

#### Enzymatic assay

The enzymatic assays of purified recombinant membrane bound *Dv*CH3H were performed in triplicates with, apigenin, kaempferol, and naringenin ((2*R*)-, (2*S*)-, and racemic) as substrates. (2*S*)-naringenin and (2*R*)-naringenin were purchased from PlantMetaChem (Marburg, Germany). Apigenin, and kaempferol were obtained from Extrasynthese (Genay, France). (+ /−)-Naringenin and NADPH were purchased from Sigma-Aldrich (Vienna, Austria). The reaction mixtures contained 5 µl purified recombinant *Dv*CH3H (50 mM Tris, 100 mM NaCl, 10% glycerol, 2 mM DDM, 0.5 mM TCEP, pH 8.0), 40 µL *Saccharomyces cerevisiae* INVSc1 microsomal preparation of recombinantly produced *Cr*CPR, 1.55 mM NADPH, 10 µM substrate in 100 mM HEPES (4- (2- hydroxyethyl)-1-piperazineethanesulfonic acid), 200 mM NaCl, pH 7.5 in a final volume of 100 µL. After 30 min reaction time at 30 °C the reaction was stopped with 20 µL of 20% acetic acid in acetonitrile. After centrifugation at 16,000 × g for 5 min the reaction solution was filtered through a 0.22 µm PTFE membrane. For analysis, a Dionex UltiMate 3000 RSLC System (Thermo Scientific) equipped with a diode array detector (DAD) was used. The product identification was carried out by comparing the retention time as well as the characteristic spectral absorption of the corresponding standards (for more details see SI and Supplementary Fig. S5).

#### Product identification by LC–MS–MS

Enzymatic assays were performed as described before, except the reactions were stopped by mixing with 10 µL acetic acid and extracted with 70 µL ethyl acetate. After drying, the reaction mixture was dissolved in 40 µL methanol.

The reaction products luteolin, eriodictyol and quercetin were identified by high-performance liquid chromatography coupled to mass spectrometry (for more details see SI, Supplementary Table S3 and Supplementary Fig. S6).

#### Kinetic analysis

Experiments for determination of kinetic parameters of recombinant *Dv*CH3H were performed by varying the substrate concentration. The amount of enzyme used was 1.25 ng for apigenin and kaempferol and 0.83 ng for racemic naringenin, as well as for (2*S*)- and (2*R*)- naringenin. Data analysis was carried out by nonlinear regression, mean values and standard deviations were calculated based on three replicates. Calculations and the graphs were prepared by employing the program OriginPro 2018 (OriginLab).

### Ethical approval

The authors confirm that the cDNA clones were obtained from a commercially available Dahlia cultivar. As such, no permissions or licences were required under institutional, national or international guidelines or regulations. The study is in full compliance with the IUCN Policy Statement on Research Involving Species at Risk of Extinction and the Convention on the Trade in Endangered Species of Wild Fauna and Flora, since the commercial varieties used are neither endangered nor at risk of extinction. The cultivar used is listed in the manuscript, including its commercial availability.

## Supplementary Information

Supplementary Information.

## Data Availability

The generated and analyzed data during the current study is supplied in this manuscript and is readily available from the corresponding authors upon reasonable request.
