# Supplementary material for: First purified recombinant CYP75B including transmembrane helix with unexpected high substrate specificity to (2R)-naringenin
Source: Sci Rep. 2022 May 20;12:8548. doi: 10.1038/s41598-022-11556-3 (PMC9122903; doi:10.1038/s41598-022-11556-3)
Supplement: Supplementary file 1 — Supplementary Information. [file 41598_2022_11556_MOESM1_ESM.pdf]

Supplementary Information

First purified recombinant CYP75B including transmembrane helix with unexpected high substrate specificity to (2*R*)-Naringenin

Johanna Hausjell[1*], Julia Weissensteiner[2*], Christian Molitor[2], Karin Schlangen[2], Oliver Spadiut[1**] and Heidi Halbwirth[2**]

[1]Technische Universität Wien, Institute of Chemical, Environmental and Bioscience Engineering, Gumpendorfer Straße 1a, 1060 Vienna, Austria

[2]Technische Universität Wien, Institute of Chemical, Environmental and Bioscience Engineering, Getreidemarkt 9, 1060 Vienna, Austria

[*]Contributed equally to the results

[**]Correspondence Bioprocessing: Oliver Spadiut, TU Wien, Institute of Chemical, Environmental and Bioscience Engineering, Research Division Biochemical Engineering, Vienna, Austria, tel.: +43 1 58801 166473; fax: +43 1 58801 166980; e-mail: oliver.spadiut@tuwien.ac.at

[**]Correspondence Biochemistry: Heidi Halbwirth TU Wien, Institute of Chemical, Environmental and Bioscience Engineering, Research Division Bioresources and Plant Science, Vienna, Austria, tel.: +43 1 58801 166559; e-mail: heidrun.halbwirt@tuwien.ac.at

Materials and Methods

Cloning and recombinant expression of *Dv*CH3H in *Pichia pastoris*

Extraction of mRNA was performed with the µMACS mRNA isolation kit (Miltenyi Biotec, Bergisch Gladbach, Germany). Reverse transcription of the obtained mRNA was achieved with the RevertAid H Minus MuLV reverse transcriptase (Fermentas Life Science, St. Leon-Rot, Germany) and the oligo(-dT) anchor primer GACCACGCGTATCGATGTCGAC$(T)_{16}$V. For the amplification of a CH3H fragment, degenerated primers designed from the 5'-noncoding region of *Cosmos sulphureus* CH3H (GenBank accession no. FJ216429) were used, in combination with the anchor primer[1]. Final proofreading amplification was carried out with specific primers designed from the obtained cDNA fragments using a Taq/Pwo polymerase system (Invitrogen, Carlsbad, CA, United States of America). Sequences were confirmed by a commercial supplier (Eurofins MWG Operon, Ebersberg, Germany). A codon optimized gene coding for *Dv*CH3H was synthesized by a commercial supplier (Genscript, Piscataway, NJ, USA). All used primers are listed in Supplementary Table S1. The cloning into the appropriate vectors, the transformation into *Pichia pastoris*, as well as the recombinant expression, was performed as described previously[2]. The cultivated cells were harvested by centrifugation at 7,000 x g for 20 min at 4 °C, the supernatant was discarded, and the cell biomass was frozen at -20 °C until further use.

Cloning and heterologous expression of *Cr*CPR

The gene encoding a NADPH-dependent cytochrome P450 reductase from *Catharanthus roseus* (*Cr*CPR) (GenBank: X69791.1) was codon-optimized for expression in *Escherichia coli* by a commercial supplier (Genscript, Piscataway, NJ, USA). The expression in *Saccharomyces cerevisiae* showed the best results with the *E. coli* codon-optimized sequence compared with the original sequence. Cloning primers (Supplementary Table S1) were derived by using the StarPrimer D´Signer software (IBA Lifesciences, Version 3.0.0.3). PR-PCR was performed with *Pfu* DNA Polymerase (Thermo Scientific) and the primer combinations *Cr*CPR-F and *Cr*CPR-R. StarGate® cloning and expression system (IBA Lifesciences) was used according to the manufacturer´s instructions (protocol version PR26-0023). In brief, PR-PCR products were inserted into pENTRY-IB51 to generate the donor vector. The insert of the donor vector was further subcloned in the acceptor vector pYSG-IBA-103 for heterologous expression in *Saccharomyces cerevisiae* strain INV*Sc*1 (Invitrogen), which allows the heterologous expression of the respective cDNAs in *Saccharomyces cerevisiae* as fusion proteins with a C-terminal Twin-Strep-Tag® (tandem peptide WSHPQFEK with an internal linker region). The heterologous expression and the microsome preparation were based on Hutabarat O.S., *et al.*[3]. In brief, an overnight culture of a single colony carrying the pYSG-IBA-103-CPR plasmid was prepared in 50 mL SGI medium and incubated at 28 °C for 24 h at 200 rpm. 250 mL YPGE medium with 20 g/L glucose were inoculated with the appropriate volume of starter culture to obtain an OD of 0.2 and incubated at 28 °C until an OD of 0.8 to 1.2 was reached. The expression was induced by adding $CuSO_4$ to the final concentration of 0.5 mM. The culture was incubated for 12 to 15 h at 28 °C for 24 h at 200 rpm. The yeast culture was centrifuged at 5,000 x g for 10 min and the pellet was resuspended in 30 mL of TEK buffer (50 mM Tris/HCl, pH 7.4, 1 mM EDTA, 0.1 M KCl) and incubated at room temperature for 5 min. The cells were recovered by centrifugation and resuspended in 2.5 mL TES-B* buffer (50 mM Tris/HCl, pH 7.4, 1 mM EDTA, 0.6 M Sorbitol, 2 mM DTT). 7.5 g of acid-washed glass beads (0.4 – 0.6 mm diameter) were added to the cell suspension, and the cell walls were mechanically disrupted by hand shaking for 20 mins, 20 times 30 s shaking and 30 s incubating on ice. The crude extract was diluted with 5 mL of TES-B* buffer, mixed and centrifuged at 160 g to separate the glass beads. This procedure was repeated twice. The pooled supernatant was centrifuged at 31,000 x g at 4 °C for 10 min. After centrifugation, the supernatant was diluted to 25 mL with TES-B*

and NaCl was added to a final concentration of 0.15 M. The microsomes were precipitated with a final concentration of 10% (w/v) polyethylene glycol (PEG)-4000 and incubated on ice for 15 min, followed by centrifugation at 13,000 x g at 4 °C for 10 min. The supernatant was discarded and the pellet was washed with 2 mL TES-B* buffer, before it was resuspended in 1.5 mL TEG-buffer (50 mM Tris/HCl, pH 7.4, 1 mM EDTA, 2 mM DTT and 20% (v/v) Glycerol). The microsomal preparations were frozen at -80 °C until further use.

Purification of transmembrane helix possessing *Dv*CH3H

Preparative Size Exclusion Chromatography. The collected eluate (2 mL) was loaded onto a pre-equilibrated HiLoad 16/600 Superdex 200 pg (GE Boston, Massachusetts, USA) for further purification. The flowrate was set to 30 cm/h. Eluting peaks were checked on a western blot, analytical SEC, and also analyzed regarding enzymatic *Dv*CH3H activity.

Analytical Size Exclusion Chromatography. Analytical size exclusion chromatography was carried out on an Ultimate HPLC 5000 (Thermo Scientific, Waltham, MA, USA) with a Superdex 200 Increase 5/150 GL (GE Boston, MA, USA) in buffer A with 2 mM DDM at a flowrate of 90 cm/h. 20 µl sample volumes were injected.

SDS-PAGE and Western Blot. Samples were incubated 1:2 with a 2 x concentrated Lämmli buffer[4] (16) at 95 °C for 10 minutes. They were loaded onto an Any kD™ Mini-PROTEAN® TGX™ Precast Protein Gel, 10-well, 30 µl (Bio-Rad, Vienna, Austria). Gels were run in SDS buffer in a Mini-PROTEAN® Tetra Vertical Electrophoresis Cell (Bio-Rad, Hercules, CA, USA) at a constant voltage of 180 V for 35 min.

The gel was blotted with 350 mA for 100 minutes onto a nitrocellulose blotting membrane (Amersham™ Protran® 0.2µm NC, GE, Boston, MA, USA). The membrane was blocked (ROTI®-BLOCK, Carl Roth, Germany) at 4 °C over night. After washing, the membrane was incubated with an Anti-His-HRP antibody (MACS Miltenyi Biotec) for 2 hours, washed again and incubated for 5 min with SuperSignal™ West Pico PLUS Chemiluminescent Substrate (Thermo Scientific, Waltham, MA, USA). A Gel Doc XR system and the ImageLab software (Bio-Rad, Hercules, CA, USA) were used for detection and quantification.

Protein Concentration. The protein concentrations were measured by Bradford assays in crude samples. Bradford reagent was purchased from Sigma Aldrich (Sigma-Aldrich, Vienna, Austria) and bovine serum albumin was used as standard. Samples were diluted with buffer A in order to be within the linear range (0.1-0.8 absorption units) of the Genesys 20 photometer (Thermo Scientific, Waltham, MA, USA).

Calculation of the overall purification process steps. The enzyme activity could not be detected in the presence of 20 mM DDM or 500 mM imidazole, hence the specific activity was estimated from the purification steps before the solubilisation. The progress of the purification process after solubilization was followed with measurements of the total protein concentration via Bradford assay. The recovery was determined by western blot, the purity was calculated from a SEC HPLC chromatogram and the specific activity was then calculated based on measurements of the purified enzyme.

Enzymatic assay. The enzymatic assays of purified recombinant membrane bound *Dv*CH3H were performed in triplicates with isoliquiritigenin, apigenin, kaempferol, and naringenin ((2*R*)-, (2*S*)-, and racemic) as substrates. (2*S*)-naringenin and (2*R*)-naringenin were purchased from PlantMetaChem (Marburg, Germany). Apigenin, isoliquiritigenin and kaempferol were obtained from Extrasynthese (Genay, France). (+/-)-Naringenin and NADPH were purchased from Sigma-Aldrich (Vienna, Austria). The reaction mixtures contained 5 µl purified recombinant *Dv*CH3H (50 mM Tris, 100 mM NaCl, 10% glycerol, 2 mM DDM, 0.5 mM TCEP, pH 8.0), 40 µL *Saccharomyces cerevisiae* INVSc1 microsomal preparation of

recombinantly produced *Cr*CPR, 1.55 mM NADPH, 10 µM substrate in 100 mM HEPES (4- (2- hydroxyethyl)-1-piperazineethanesulfonic acid), 200 mM NaCl, pH 7.5 in a final volume of 100 µL. After 30 min reaction time at 30 °C, the reaction was stopped with 20 µL of 20% acetic acid in acetonitrile. After centrifugation at 16,000 x g for 5 min the reaction solution was filtered through a 0.22 µm PTFE membrane. For analysis, a Dionex UltiMate 3000 RSLC System (Thermo Scientific) equipped with a diode array detector (DAD) was used. A 40 µL aliquot was loaded on an Acclaim RSLC 120 C18 column. The mobile phases were (A) H_2O with 0.1% formic acid and (B) MeCN with 0.1% formic acid. The flow rate was set to 0.2 mL/min, and the column temperature was set to 25 °C.

The gradient for the reaction of ((2*R*)-, (2*S*)-, and racemic) naringenin to eriodictyol was set as follows: -6−0 min, 20% B; 0−17 min, 20−95% B; 17−18 min, 95% B. Absorbance monitoring for quantification was performed at the absorbance wavelength of eriodictyol of 290 nm.

The gradient for the reaction of apigenin to luteolin and kaempferol to quercetin was set as follows: -6−0 min, 20% B; 0−13 min, 20−95% B; 13−14 min, 95% B. Monitoring for quantification was set to the absorbance wavelengths of luteolin at 346 nm and of quercetin of 370 nm, respectively.

Product identification by LC-MS-MS.

Enzymatic assays were performed as described in the main text, except the reactions were stopped by admixture of 10 µL acetic acid and extracted with 70 µL ethyl acetate. The organic solvent was removed under vacuum, the residue was resuspended in 40 µL methanol and filtrated through a 0.22 µm PTFE membrane.

The reaction products luteolin, eriodictiol and quercetin were identified by high-performance liquid chromatography coupled to mass spectrometry (HPLC-MS-MS). 4 µL of the extract were injected on a 1290 Infinity II LC System (Agilent, Santa Clara, USA) equipped with a 1260 Infinity II diode array detector (DAD). The separation was performed on a ZORBAX Eclipse Plus C18 Rapid Resolution 1.8 µm, 2.1 x 150 mm^2 column (Agilent, Santa Clara, USA) at 35 °C and 0.3 mL/min. The mobile phases were (A) H_2O with 0.1% formic acid and (B) MeCN with 0.1% formic acid. The gradient was set as follows: -7−1 min, 5% B; 1−5 min, 5−15% B; 5−11 min, 15−53% B; 11−15 min, 53−100% B; 21.5 min, 100% B.

Mass detection was performed using an Agilent High-Resolution-y MS 6545 Q-TOF with Ion Source (Agilent Technologies, Santa Clara, USA).
The main instrumental conditions were as follows: negative electrospray ionization mode, MS scan range was from m/z 100 to 1000, product ion scan range from m/z 50 to 325, capillary voltage 2.0 kV; gas temperature 350 °C; vaporizer temperature 220 °C; gas flow 5L/min, nebulizer 60 psi, fragmentor voltage 180 V; skimmer 75 V. Nitrogen was used as nebulizer and auxiliary gas. The collision energy for the fragmentation was set on 32 eV for luteolin, on 15 eV for eriodictyol and on 23 eV for quercetin.

Data acquisition was carried out using Agilent Mass Hunter Workstation Data Acquisition (AB Sciex, Foster City, USA) and evaluated using Agilent MassHunter Qualitative Analysis 10.0. Identifications were based on chromatographic elution time, Accurate Mass, MS/MS fragmentation pattern, and comparisons with available standards.

Additional Files

Supplementary Table S1: Overview of the percentage of amino acid sequence identities of the isolated CH3H of *Dahlia variabilis* and further CH3Hs and F3'Hs from Asteraceae species. CH3Hs are highlighted in bold. All other are F3'Hs. The sequence identity map was obtained with Clustal Omega.

	ACN65827	ACN65826	ABA64468	ACN65825	ABB29899	**ACO35755**	**Dahlia**	**BDE26439**	ACO35752	ADB77826	ADB77825	ACO35756	CO35757
ACN65827 *Centaurea*	100.00	80.63	79.57	80.82	81.09	76.95	77.76	77.76	80.12	81.30	81.10	79.76	80.16
ACN65826 *Echinops*	80.63	100.00	83.47	84.75	83.79	80.04	80.63	80.83	81.87	84.26	83.86	83.30	83.70
ABA64468 *Gerbera*	79.57	83.47	100.00	84.81	82.32	79.72	80.63	81.15	83.27	84.45	84.25	83.27	83.66
ACN65825 *Cichorium*	80.82	84.75	84.81	100.00	84.93	81.02	82.05	82.25	83.23	85.21	84.81	83.46	83.27
ABB29899 *Osteospermum*	81.09	83.79	82.32	84.93	100.00	82.62	82.48	82.48	84.45	86.22	85.83	85.46	85.66
**ACO35755 *Cosmos***	76.95	80.04	79.72	81.02	82.62	100.00	90.94	90.94	85.43	85.83	85.63	84.09	84.87
**GQ479804 *Dahlia* CH3H**	77.76	80.63	80.63	82.05	82.48	90.94	100.00	99.61	85.91	88.69	88.49	85.74	86.53
**BDE26439 *Dahlia***	77.76	80.83	81.15	82.25	82.48	90.94	99.61	100.00	85.91	88.49	88.29	85.54	86.53
ACO35752 *Cosmos*	80.12	81.87	83.27	83.23	84.45	85.43	85.91	85.91	100.00	89.57	89.37	87.80	88.39
ADB77826 *Dahlia* 2	81.30	84.26	84.45	85.21	86.22	85.83	88.69	88.49	89.57	100.00	99.41	89.96	90.55
ADB77825 *Dahlia* 1	81.10	83.86	84.25	84.81	85.83	85.63	88.49	88.29	89.37	99.41	100.00	89.57	90.16
ACO35756 *Tagetes*	79.76	83.30	83.27	83.46	85.46	84.09	85.74	85.54	87.80	89.96	89.57	100.00	98.04
ACO35757 *Rudbeckia*	80.16	83.70	83.66	83.27	85.66	84.87	86.53	86.53	88.39	90.55	90.16	98.04	100.00

Supplementary Table S2: List of primers. Primers used for the amplification of *DvCH3H* cDNA open reading frames as well as for the amplification of *CrCPR*

Name	Primer sequence (5’ -> 3’)
Dahl_CH3H.for	ATGTCTATTCTACCCCTACTACTTTACC
Dahl_CH3H.rev	CTATAACATTTGAAACAGGAACTCGAGTT
CrCPR-for	AGCGGCTCTTCAATGGACAGCAGCAGCGAGAAGC
CrCPR-rev	AGCGGCTCTTCTCCCCCACACGTCACGCAGATAAC

Supplementary Table S3: Mass spectrometric data.

Analytes	Theoretical precursor ion [m/z]	Measured precursor ion [m/z]	Δppm	base peak ion [m/z]	Collision energy [eV]
Luteolin	285.0405	285.0407	0.7017	133 (100)	32
Eriodictyol	287.0561	287.0556	-1.7418	151 (100)	15
Quercetin	301.0354	301.0351	-0.9966	151 (100)	23

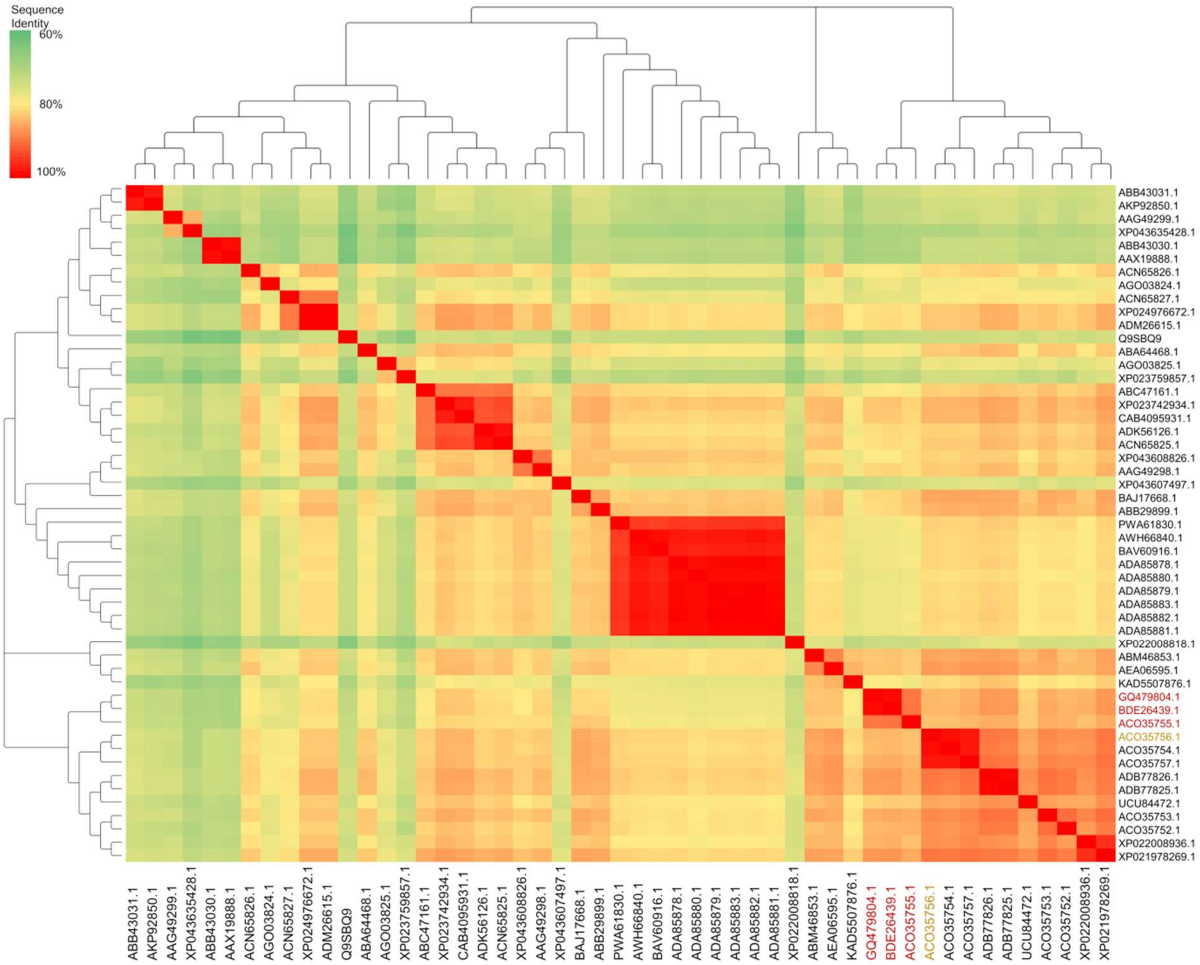

Supplementary Figure S1: Sequence identity matrix map of Asteraceae CYP75B enzymes. The sequence identity values were obtained by pairwise alignments using MatGAT[5] and by applying the BLOSUM62 algorithm. The sequences are ordered identical to the phylogenetic tree in the main text.

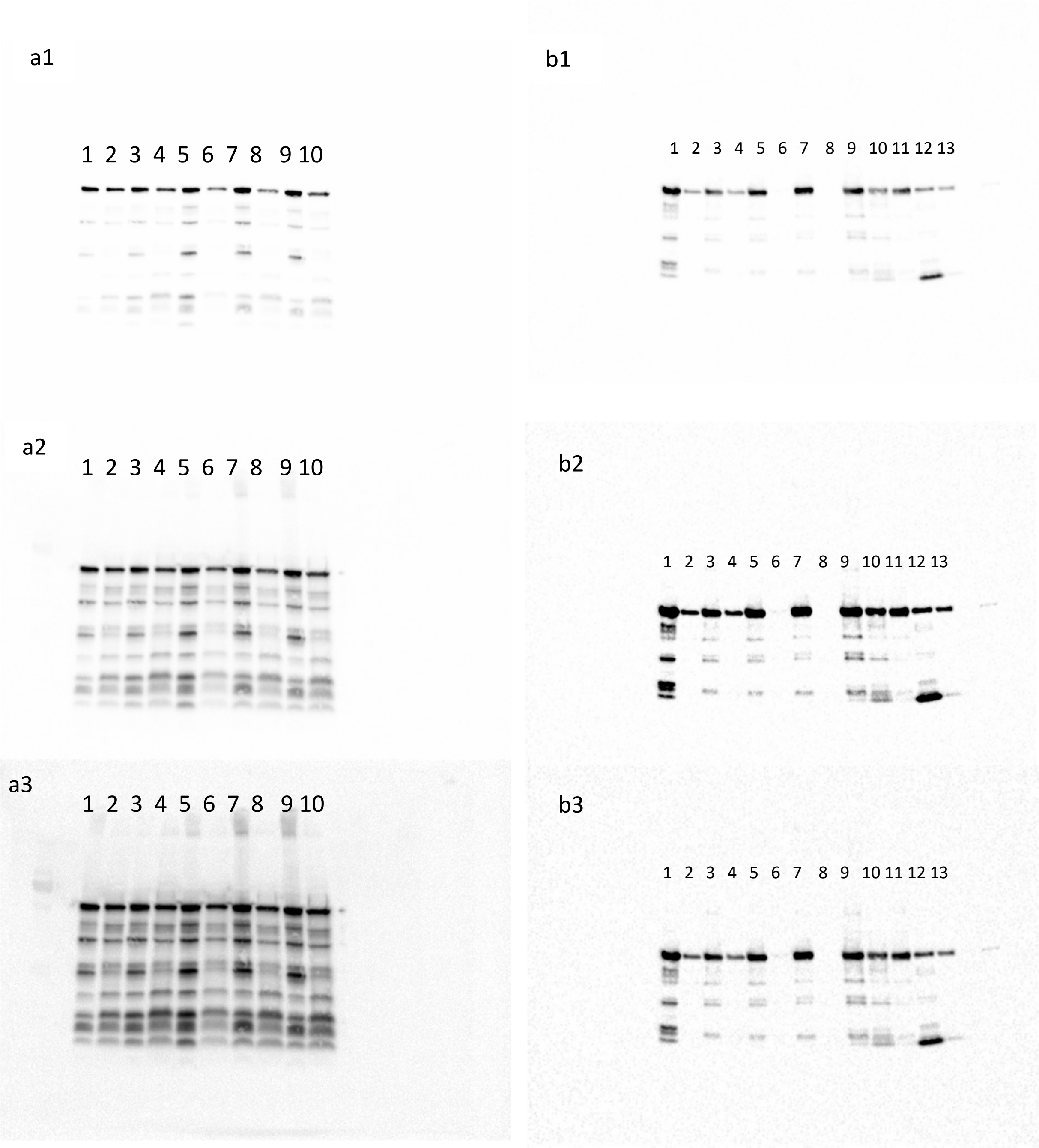

Supplementary Figure S2: Western blots of fractions during purification of *Dv*CH3H. (a1-3) Supernatants and pellets of yeast biomass centrifuged at different biomass concentrations; 1: 6 g/L DCW Pellet, 2: 6 g/L DCW Supernatant, 3: 19.5 g/L DCW Pellet, 4: 19.5 g/L DCW Supernatant, 5: 33 g/L DCW Pellet, 6: 33 g/L DCW Supernatant, 7: 46.5 g/L DCW Pellet, 8: 46.5 g/L DCW Supernatant, 9: 60 g/L DCW Pellet, 10: 60 g/L DCW Supernatant. (b1-3) Supernatants and pellets after solubilisation of the membrane in different detergents; 1: Start, 2: DDM Supernatant, 3: DDM Pellet, 4: DM Supernatant, 5: DM Pellet, 6: CHAPS Supernatant, 7: CHAPS Pellet, 8 FC-12 Supernatant, 9: FC Pellet, 10: LDAO Supernatant, 11: LDAO Pellet, 12: OTG Supernatant, 13: OTG Pellet. Please note: a1-3 and b1-3 present the respective blots at different contrast settings.

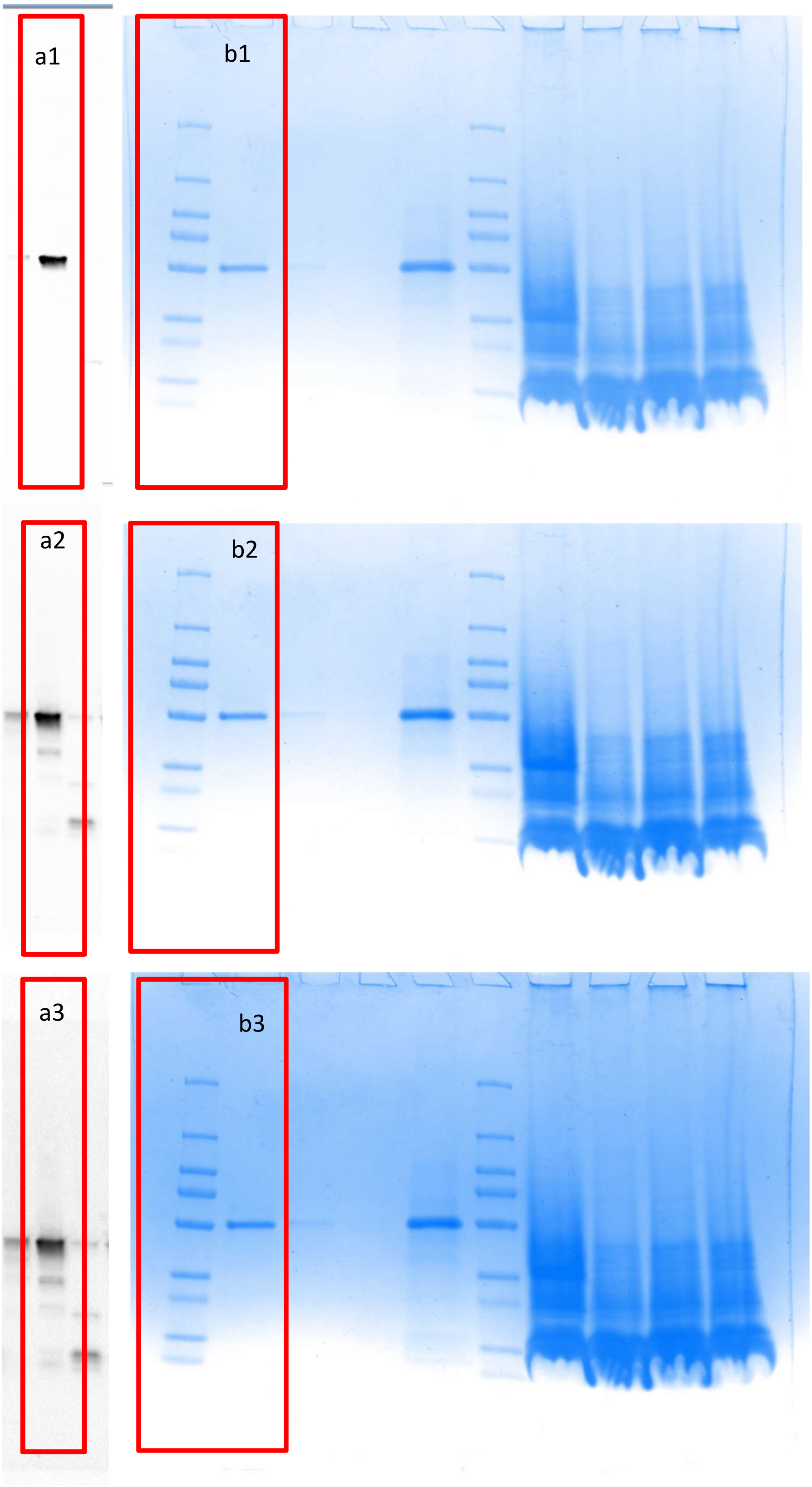

Supplementary Figure S3: Originals of figure 5, showing western blot (a) and SDS-PAGE (b) of the purified recombinant *Dv*CH3H. Please note that only the lanes outlined in red are relevant to this article.

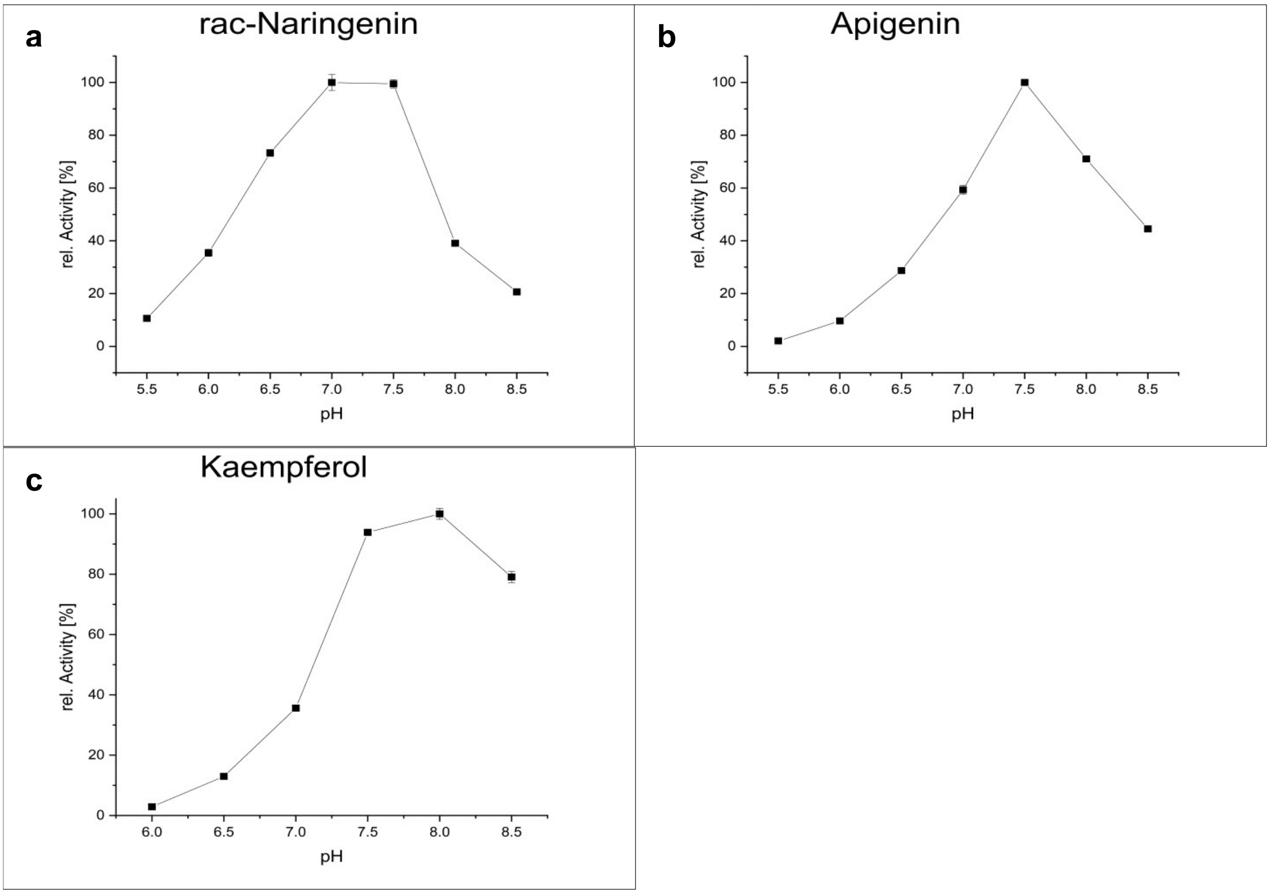

Supplementary Figure S4: pH Optimum of the membrane bound *Dv*CH3H. The pH optimum was investigated with 1 µM naringenin (A), 2.5 µM apigenin (B) and 10 µM kaempferol (C) as substrate. The substrates in 100 mM MES buffer (2-(N-morpholino) acid) were used over a range from pH 5.5 to 6.5 , in 100 mM HEPEs buffer from pH 7.0 to 7.5 and in 100 mM Tris buffer (tris(hydroxymethyl)aminomethane) from pH 8.0 to 8.5. The highest enzyme activity at optimal pH was set to 100%.

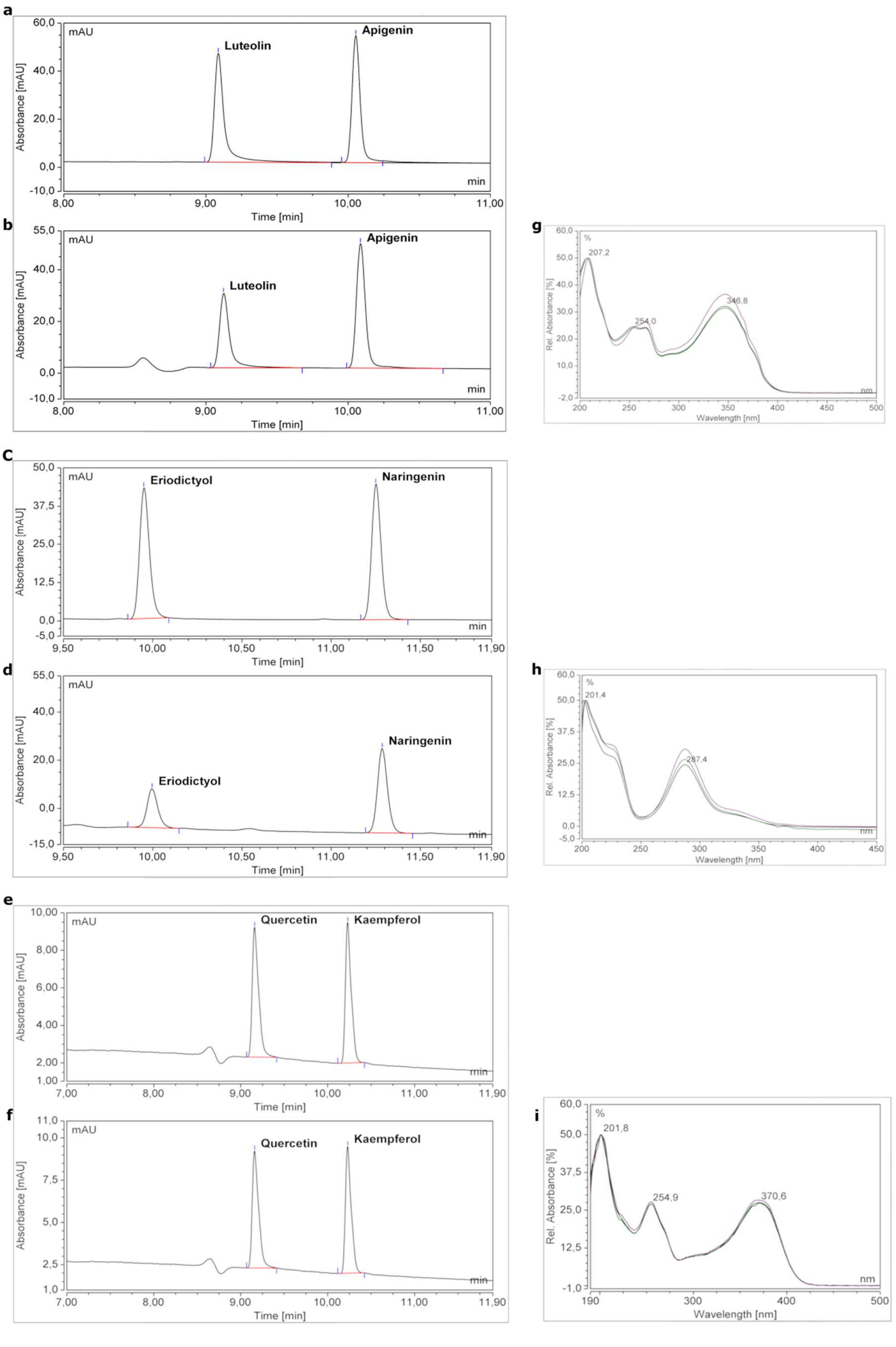

Supplementary Figure S5: HPLC Chromatograms with the appropriate UV/vis spectra of the products. (a) Standard substates apigenin and luteolin. (b) Reaction of apigenin to luteolin catalysed by *Dv*CH3H. (c) Standard naringenin and eriodictyol. (c) Reaction of naringenin to eriodictyol catalysed by *Dv*CH3H. (e) Standard kaempferol and quercetin. (f) Reaction of kaempferol to quercetin catalysed by *Dv*CH3H. (g) UV/vis Spectra of luteolin catalysed by *Dv*CH3H. (h) UV/vis Spectra of eriodictyol catalysed by *Dv*CH3H. (i) Uv/vis Spectra of quercetin catalysed by *Dv*CH3H.

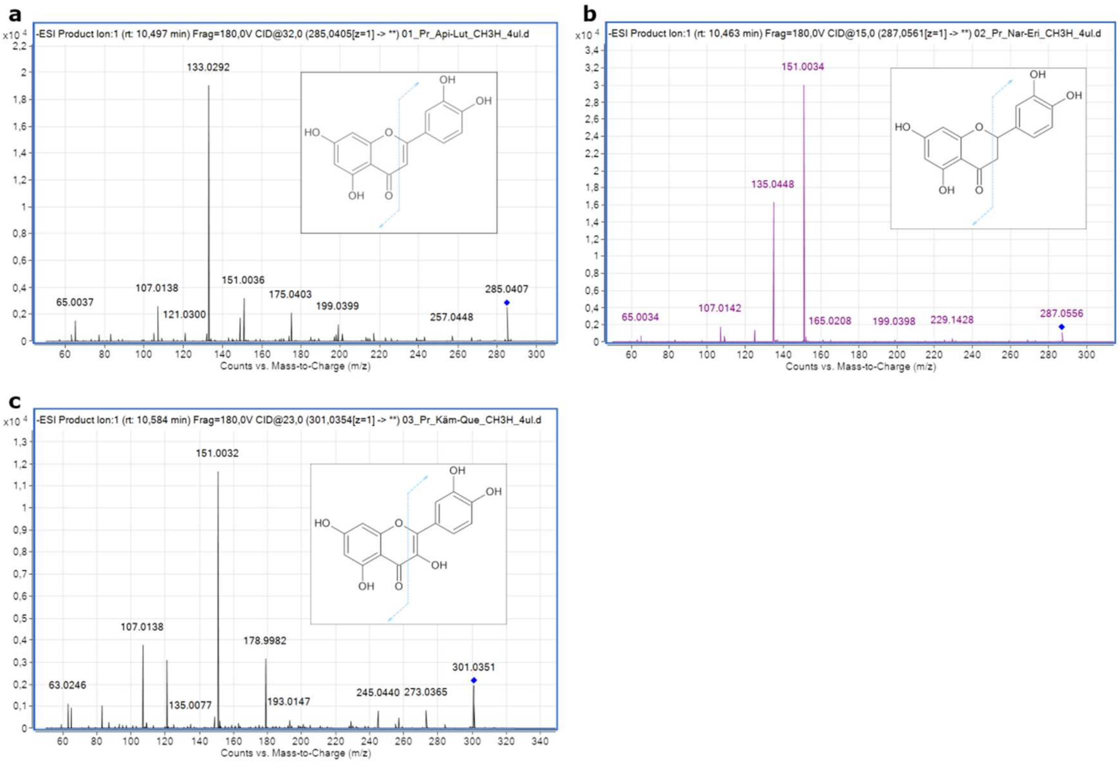

Supplementary Figure S6: Product ion spectra of luteolin (a), eriodictyol (b) and quercetin (c). The mass of the precursor ion is market by a blue dot. The fragmentation patterns of the enzymatic reaction products are identical to the respective standard.

References

1 Wimmer, G., Halbwirth, H., Wurst, F., Forkmann, G. & Stich, K. Enzymatic hydroxylation of 6′-deoxychalcones with protein preparations from petals of Dahlia variabilis. *Phytochemistry* **47**, 1013-1016, doi:https://doi.org/10.1016/S0031-9422(98)80063-0 (1998).

2 Hausjell, J. *et al.* Recombinant production of a hard-to-express membrane-bound cytochrome P450 in different yeasts-Comparison of physiology and productivity. *Yeast* **37**, 217-226, doi:10.1002/yea.3441 (2020).

3 Hutabarat, O. S. *et al.* Transgenic apple plants overexpressing the chalcone 3-hydroxylase gene of Cosmos sulphureus show increased levels of 3-hydroxyphloridzin and reduced susceptibility to apple scab and fire blight. *Planta* **243**, 1213-1224, doi:10.1007/s00425-016-2475-9 (2016).

4 Laemmli, U. K. Cleavage of structural proteins during the assembly of the head of bacteriophage T4. *Nature* **227**, 680-685, doi:10.1038/227680a0 (1970).

5 Campanella, J. J., Bitincka, L. & Smalley, J. MatGAT: an application that generates similarity/identity matrices using protein or DNA sequences. *BMC Bioinformatics* **4**, 29, doi:10.1186/1471-2105-4-29 (2003).
